# The effects of the primary health care providers’ prescription behavior interventions to improve the rational use of antibiotics: a systematic review

**DOI:** 10.1186/s41256-020-00171-2

**Published:** 2020-10-17

**Authors:** Lu Yao, Jia Yin, Ruiting Huo, Ding Yang, Liyan Shen, Shuqin Wen, Qiang Sun

**Affiliations:** 1 Centre for Health Management and Policy Research, School of Public Health, Cheeloo College of Medicine, Shandong University, Jinan, 250012 China; 2 NHC Key Lab of Health Economics and Policy Research (Shandong University), Jinan, 250012 China; 3 Cangzhou Central Hospital, Cangzhou, 061001 Hebei China

## Abstract

**Background:**

Irrational antibiotics use in clinical prescription, especially in primary health care (PHC) is accelerating the spread of antibiotics resistance (ABR) around the world. It may be greatly useful to improve the rational use of antibiotics by effectively intervening providers’ prescription behaviors in PHC. This study aimed to systematically review the interventions targeted to providers’ prescription behaviors in PHC and its’ effects on improving the rational use of antibiotics.

**Methods:**

The literatures were searched in Ovid Medline, Web of Science, PubMed, Cochrane Library, and two Chinese databases with a time limit from January 1st, 1998 to December 1st, 2018. The articles included in our review were randomized control trial, controlled before-and-after studies and interrupted time series, and the main outcomes measured in these articles were providers’ prescription behaviors. The Cochrane Collaboration criteria were used to assess the risk of bias of the studies by two reviewers. Narrative analysis was performed to analyze the effect size of interventions.

**Results:**

A total of 4422 studies were identified in this study and 17 of them were included in the review. Among 17 included studies, 13 studies were conducted in the Europe or in the United States, and the rest were conducted in low-income and-middle-income countries (LMICs). According to the Cochrane Collaboration criteria, 12 studies had high risk of bias and 5 studies had medium risk of bias. There was moderate-strength evidence that interventions targeted to improve the providers’ prescription behaviors in PHC decreased the antibiotics prescribing and improved the rational use of antibiotics.

**Conclusions:**

Interventions targeted PHC providers’ prescription behaviours could be an effective way to decrease the use of antibiotics in PHC and to promote the rational use of antibiotics. However, we cannot compare the effects between different interventions because of heterogeneity of interventions and outcome measures.

## Background

Antibiotics resistance (ABR) is a growing public health problem [1], which delayed the therapy effectiveness, greatly increasing the health costs and the risk of morbidity and mortality [2]. In 1998, the World Health Assembly initially issued a separate motion on ABR and put forward a comprehensive management proposal to respond to the emergence of ABR [3]. In the same year, the World Health Organization (WHO) advocated to cope with the ABR crisis by promoting the rational use of antibiotics globally [4].

The healthcare providers’ prescribing behaviours is an important area to promote the rational use of antibiotics. The previous studies have shown that many countries have been successful in reducing prescribing of antimicrobials in secondary and tertiary hospitals in the past decades. However, irrational use of antibiotics in primary health care (PHC) was still problematic, and especially in the context when a large majority of people are prescribed with antibiotics [5, 6]. It is estimated that about 80% antimicrobials were consumed in PHC around the world [7]. Therefore, effetive interventions to improve healthcare providers’ prescribing behaviours in PHC would greatly improve the rational use of antibiotics.

A Cochrane review in 2017 examined the effectiveness of interventions on health professionals’ antibiotics prescribing practices for hospital inpatients, and it was observed that antimicrobial stewardship interventions can greatly reduce unnecessary antibiotics use in hospital settings [8]. These interventions are typically classified as educational intervention, audit and feedback interventions, health policy change strategies, as well as organizational or professional financial incentives to improve the quality of antibiotics prescribing [8]. Several reviews has also reported positive effects of hospital antibiotics stewardship interventions. And there are types of interventions, structure interventions such as new technology for rapid microbiology testing or measurement of inflammatory markers, persuasive interventions like expert audit of prescriptions and feedback advice to prescribers, enabling interventions like guidelines or education on antibiotics use and restrictive interventions like expert approval for use of certain antibiotics) [9, 10].

Preliminary analysis suggests that very few studies are conducted to explore the effectiveness of interventions to decrease antibiotics prescribing and to promote the antibiotics prescribing behaviours from the perspective of primary health providers. This also necessities to have a thorough analysis of the issue. In this context the current systematic review is planned. The objective of the review is to conduct a systematic review of literature to evaluate the effects of the primary health care providers’ prescription behavior interventions in improving the rational use of antibiotics.

## Methods

The review protocol of this study, with the search strategy included, was registered at the PROSPERO international prospective register of systematic reviews (CRD:42019146631).

### Search strategy

We searched the following databases from January 1st, 1998 to December 1st, 2018: The databases include Ovid Medline, Web of Science, PubMed and Cochrane Library were searched for relevant studies published in English, and the databases of China National Knowledge Infrastructure (CNKI) and WANFANG database were searched for Chinese language studies. The Chinese databases were searched using the following terms (in Chinese): ‘prescription’, ‘community’, ‘primary health’, ‘outpatient’, ‘rural doctors’, ‘village doctors’ ‘intervention’, ‘antimicrobial’, ‘antibacterial’ and ‘antibiotics’. The search strategies used to search at Ovid Medline, Web of Science, PubMed and Cochrane Library can be seen in the Supplemental documents. Identification of relevant studies was carried out by one researcher and checked by two other researchers. Additional studies were identified by cross-referencing. The experts were also consulted for additional literature. The flow chart of the searching was referred to the PRISMA protocol.

### Inclusion and exclusion criteria

We included studies according to PICOS (population, intervention, comparison, outcome and study design) characteristic. Population: the participants refer to the physicians at outpatient clinics, general practitioners, rural doctors; the patients included were not specified (e.g. respiratory tract infections or urinary tract infections). Intervention: the studies regarding promoting the antibiotics rational use and the interventions targeted towards primary health care providers. We referred EPOC (Cochrane Effective Practice and Organisation of Care) taxonomy (EPOC 2015) [11] to include interventions of educational, audit and feedback, reminders and health policies changes. Outcomes: the primary outcome were the changes in antibiotics prescribing behaviors of providers, including the changes in antibiotics prescribing rates, the odds ratio of antibiotics prescribing, the percentage of prescriptions of specific antibiotics or prescribing appropriateness. Study design: the design of the studies had to be RCTs (randomized controlled trials), ITS (interrupted times series) or controlled before-and-after studies.

Articles were excluded if they focused on microbiology; were non-research articles such as reviews, meeting reports, policy briefs; or did not focus on outpatient antibiotics prescriptions. Titles and abstracts were independently screened for eligibility by two authors. In order to maintain agreement, the two researchers evaluated the quality of studies by reading the full-text articles.

### Data extraction and analysis

We used the data extracted forms from the Cochrane Handbook for Systematic Reviews [12]. The following information was extracted from each included article: first author and year of publication, study design, setting, country, participants, intervention details, target illness, duration and outcomes measures. Narrative synthesis was used due to the great heterogeneity among the included studies.

### Quality assessment

We assessed the risk of bias based on the Cochrane Collaboration criteria (Higgins 2011) [12]. We used eight standard criteria for RCTs: random sequence generation, allocation concealment, blinding of participants, blinding of outcome assessment, incomplete outcome data, selective reporting and no risk of bias from other sources. We used two additional criteria that the EPOC (Cochrane Effective Practice and Organization of Care) Group specifies (EPOC 2009): baseline characteristic similarity, as well as “adequate protection against contamination”.

We used seven criteria for NRT (non-randomized trial): the intervention is independent of other changes, the shape of the intervention effect is pre-specified, the intervention is unlikely to affect data collection, knowledge of the allocated interventions is adequately prevented during the study, the outcome data are incomplete, selective reporting, and other bias.

The Cochrane Collaboration criteria was used to assessed the risk of bias of the studies by two reviewers. The disagreements between reviewers’ judgements were resolved by discussion and consensus.

## Results

**Fig. 1:**
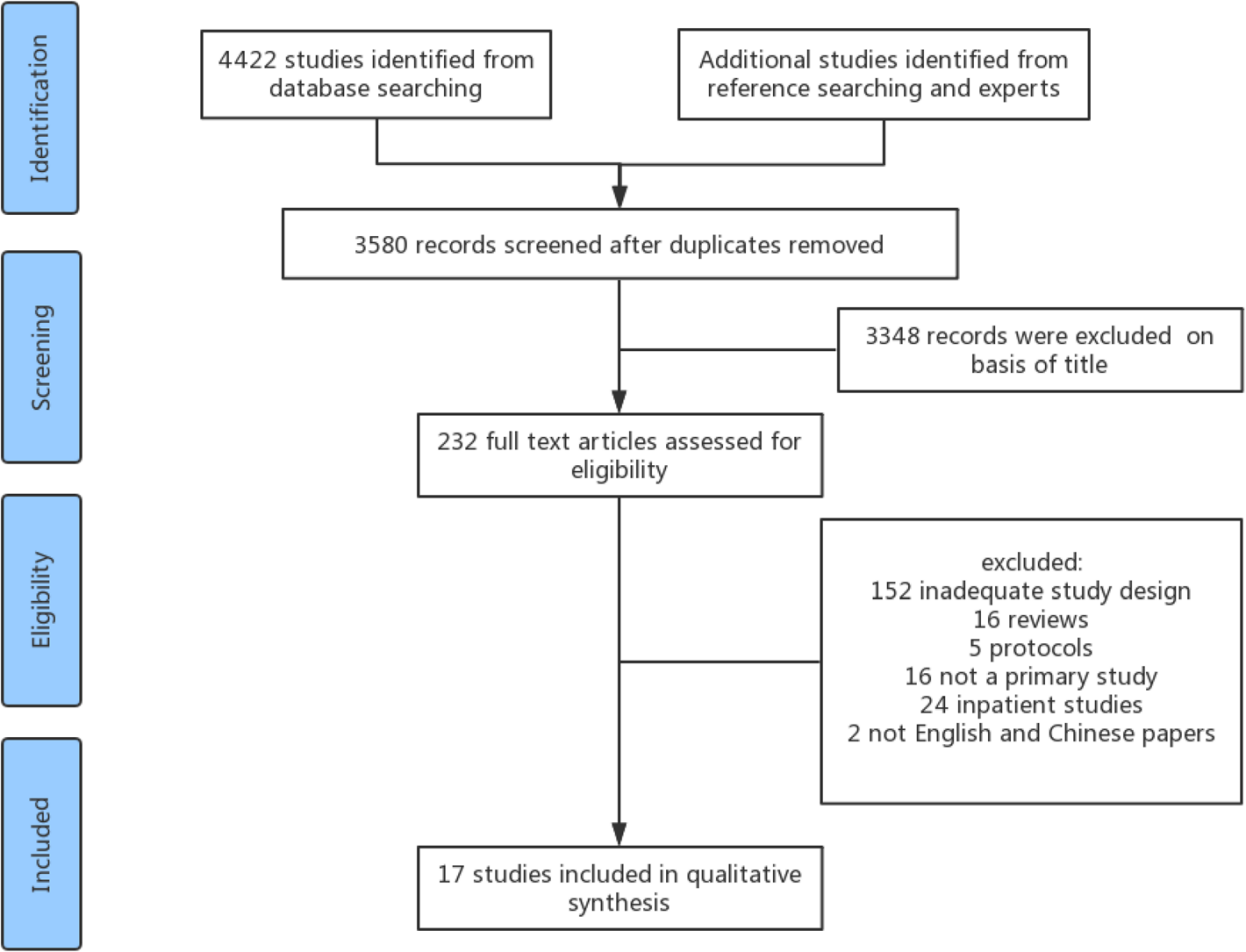
Flow diagram of systematic review screening

The process of study identification and inclusion is shown in Fig. 1. A total of 4422 articles were identified as relevant. After reviewing the abstracts and full texts, 17 studies were included, of which, 16 were in English and 1 was in Chinese.

### Study characteristics

#### Population

Of the 17 studies, ten were conducted in Europe [13, 15–18, 20, 21, 25, 26, 28], four in China [14, 22–24], three in the USA [19, 27, 29]. Table 1 provides a summary of the key characteristics of each included study. All interventions were targeted at primary health care providers including general practitioners and primary health care physicians, but excluding specialist care or outpatient department in a hospital setting. These studies focus on patients who were diagnosed with respiratory tract infections, urinary tract infections, upper respiratory tract infections.

**Table 1:** Basic characteristics of included studies (*n*=17)

Study ID	Study design	Country	Participants	Setting	Intervention details	Target illness	Duration
**Educational interventions**							
Llor et al. 2014 [13]	before-after quality assurance study	Spain	General practitioners (GPs) registered all patients with RTIs for 15 days in winter 2008	Primary Care centres in Spain	Meetings with the presentation and discussion of the results, and several training meetings on RTI guidelines, workshops on point-of-care tests -rapid antigen detection tests and C-reactive protein rapid test.	RTIs	1 year
Wei et al. 2017 [14]	cluster-RCT	China	Participants attended a township hospital as an outpatient, were aged between 2 and 14 years old, and were given a prescription of upper respiratory tract infection	25 township hospitals within the rural, low-income province of Guangxi in western China	Clinical guidelines; monthly peer-review meetings, integrated within routine monthly administrative meetings, during which doctors’ antibiotic prescribing rates were assessed; we developed leaflets and a video educating caregivers about antibiotics.	Upper respiratory tract infections	6 months
Hernandez Santiago et al. 2015 [15]	interrupted time series	United Kingdom	408058 residents of the Tayside region of Scotland	Local general practices clinics	Practices received a range of educational material, specific feedback on their own use of antimicrobials; the local Antimicrobial Management Team gave specific advice to general practices	not specified	5 years
Lemiengre et al. 2018 [16]	cluster-RCT	Belgium	169 FPs started recruitment and 3288 acute infectious episodes	Clinician practices	(1) a point-of-care C-reactive protein test (POC CRP); (2) a brief intervention to elicit parental concern combined with safety net advice (BISNA); (3) both POC CRP and BISNA;	ARTIs (acute respiratory tract infections)	1 year
Hürlimann et al. 2014 [17]	cluster-RCT	Switzerland	16863 cases with RTIs and 4245 cases with lower UTIs per year	140 primary care physicians in Switzerland	Printed guidelines for antibiotic prescription in RTIs and UTIs; individual feedback on antibiotic prescribing behaviour	RTIs and UTIs	16 months
**Audit and feedback interventions**							
Altiner et al. 2007 [18]	cluster-RCT	Germany	104 GPs in North-Rhine and Westphalia-Lippe	Regional GPs clinics	GPs in the intervention group were visited by GP peers in their clinics	RTIs	6 weeks/1 year
Gerber et al. 2013 [19]	cluster-RCT	USA	162 clinicians participated.	A network of 25 pediatric primary care practices	One 1-hour on-site clinician education session (June 2010) followed by 1 year of personalized, quarterly audit and feedback of prescribing for bacterial and viral ARTIs or usual practice.	ARTIs (acute respiratory tract infections)	1 year
Welschen et al. 2004 [20]	RCT	Netherlands	patients presenting with acute symptoms of the respiratory tract	Peer review groups (general practitioners) in the region of Utrecht	Group education meetings; monitoring and feedback on prescribing behavior; group education for assistants of general practitioners and pharmacists; Education materials for patients	acute symptoms of the respiratory tract	1 year
van der Velden et al. 2016 [21]	cluster-RCT	Netherlands	169 general practitioners	88 primary care practices participating	GP education, audit/feedback and patient information	ARTI	10–12 months
**Health policy change strategies**							
Yang 2014 [22]	A matched-pair cluster-randomized trial	China	public residents in 20 participating primary care organisations	QJ city of Hubei province, involving 20 primary care organisations	Public reporting on antibiotic prescribing for URTIs	upper respiratory tract infections	1 year
Yip et al. 2014 [23]	A matched-pair cluster-randomized trial	China	twenty-eight towns centers and posts	Twenty-eight towns in Ningxia province	This study’s policy intervention changed NCMS payments to township health centers and village posts from fee-for-service to a capitated budget with pay-for-performance.	not specified	3 years
Xiaoxia 2017 [24]	control before and after	China	Heads of different departments of primary health centers	17 primary health centers in Jiande, China	Prescribing check results as an important indicator of professional promotion and bonus performance; feedback and audit on primary center doctors prescribing.	not specified	3 months
**Information system supported interventions**							
Gulliford et al. 2014 [25]	cluster-RCT	United Kingdom	Individual patients included all those aged 18 to 59 years who were registered with the trial practices.	445 family practices	The decision support tools were installed remotely at the intervention arm practices and delivered during consultations	Urinary Tract Infections	1 year
Vellinga et al. 2016 [26]	cluster-RCT	Ireland	A total of 920 patients with suspected urinary tract infection	30 eligible practices in Irish Primary Care	All practices received a workshop to promote consultation coding for urinary tract infections; a reminder integrated into the patient management software suggested first-line treatment;	urinary tract infection	14 months
Mainous et al. 2013 [27]	quasi-experimental trial	USA	27 physicians, six nurse practitioners and six physician’s assistants volunteered to participate in this study.	Nine intervention practices and 61 control practices	Quarterly EHR based audit and feedback, ‘best-practice’ dissemination during meetings of practice representatives and practice site visits for academic detailing, performance review, and CDSS training.	ARTIs	15 months
Blair 2017 [28]	cluster-RCT	England	542 Children (aged 3 months to <12 years) with acute cough and respiratory tract infection (RTI)	32 general practices’ clinics	A web-based clinician-focused clinical rule to predict risk of future hospitalisation and a printed leaflet with individualised child health information for carers, safety-netting advice and a treatment decision record.	RTIs	1 year
Meeker et al. 2016 [29]	RCT	USA	248 enrolled clinicians	47 primary care practices	suggested alternatives presented electronic order sets; accountable justification prompted clinicians to enter free-text justifications for prescribing antibiotics; peer comparison	ARTIs	18 months

#### Intervention

Diverse interventions were observed in the included studies, five of which mainly evaluated the educational interventions, i.e., educational material, guidelines, training sessions; four used audit and feedback interventions including peer review about the prescribing, monitoring and feedback on prescribing behaviors; three used health policy change strategies including public report prescriptions, changing in payments methods and including the antibiotics using into performance. The other five employed health information system supported interventions. These were related to clinical supported decision-making system and also related to providing online guideline materials.

#### Outcomes measured

The most frequently measured outcome was antibiotics prescribing rate. Thirteen studies measured a change in antibiotics prescription rate or the odds ratio of antibiotics prescribing [13, 14, 16, 18–23, 25–29]. The antibiotics prescription rate defined as the proportion of prescriptions for specific disease that include at least one antibiotic. One study measured the impact of interventions on the rate per 1000 registered patients dispensed one or more 4C antimicrobial prescriptions (co-amoxiclav, cephalosporins, fluoroquinolones and clindamycin) [15]. Another study measured the effect on prescriptions of penicillin for RTIs (respiratory tract infections) and one on proportion of prescriptions for recommended [17]. One Chinese study measured the effect on changes in types of antibioticss [24]. Akke Vellinga used the proportion of antimicrobial prescribing of guidelines for urinary tract infection to measure the changes of providers prescription behaviors [26].

#### Study design

There were nine cluster random control trials [14, 16–19, 21, 25, 26, 28], two matched-pair cluster-randomized trials [22, 23], two RCTs [20, 29], two before and after intervention studies [13, 24], one quasi-experimental trial [27] and one interrupted time series study [15].

### Risk of bias assessment

**Fig. 2:**
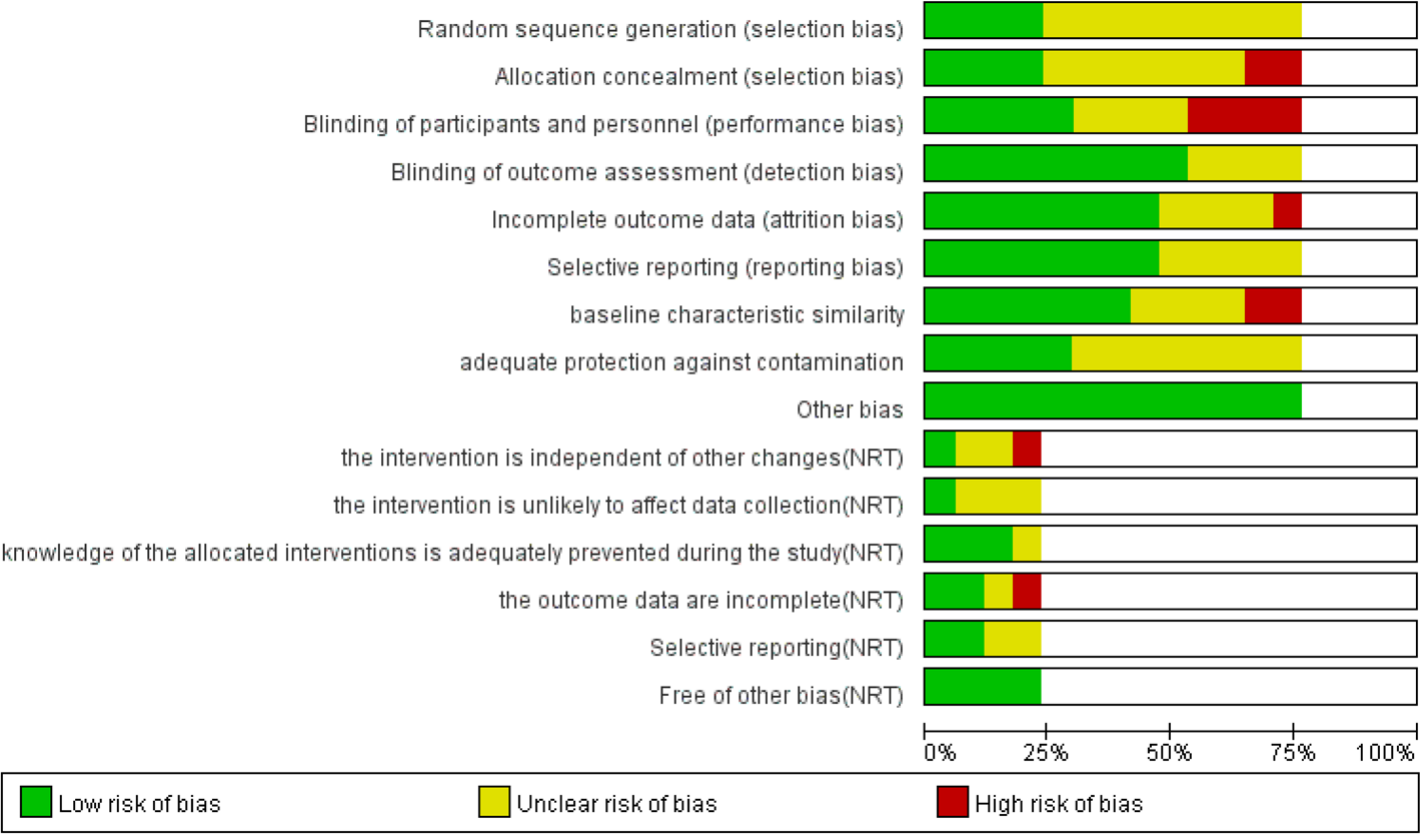
Risk of bias graph: review authors’ judgements about each risk of bias item presented as percentages across all included studies. Blank sections in this graph are due to use of different ROB criteria for RCT versus ITS studies

**Fig. 3:**
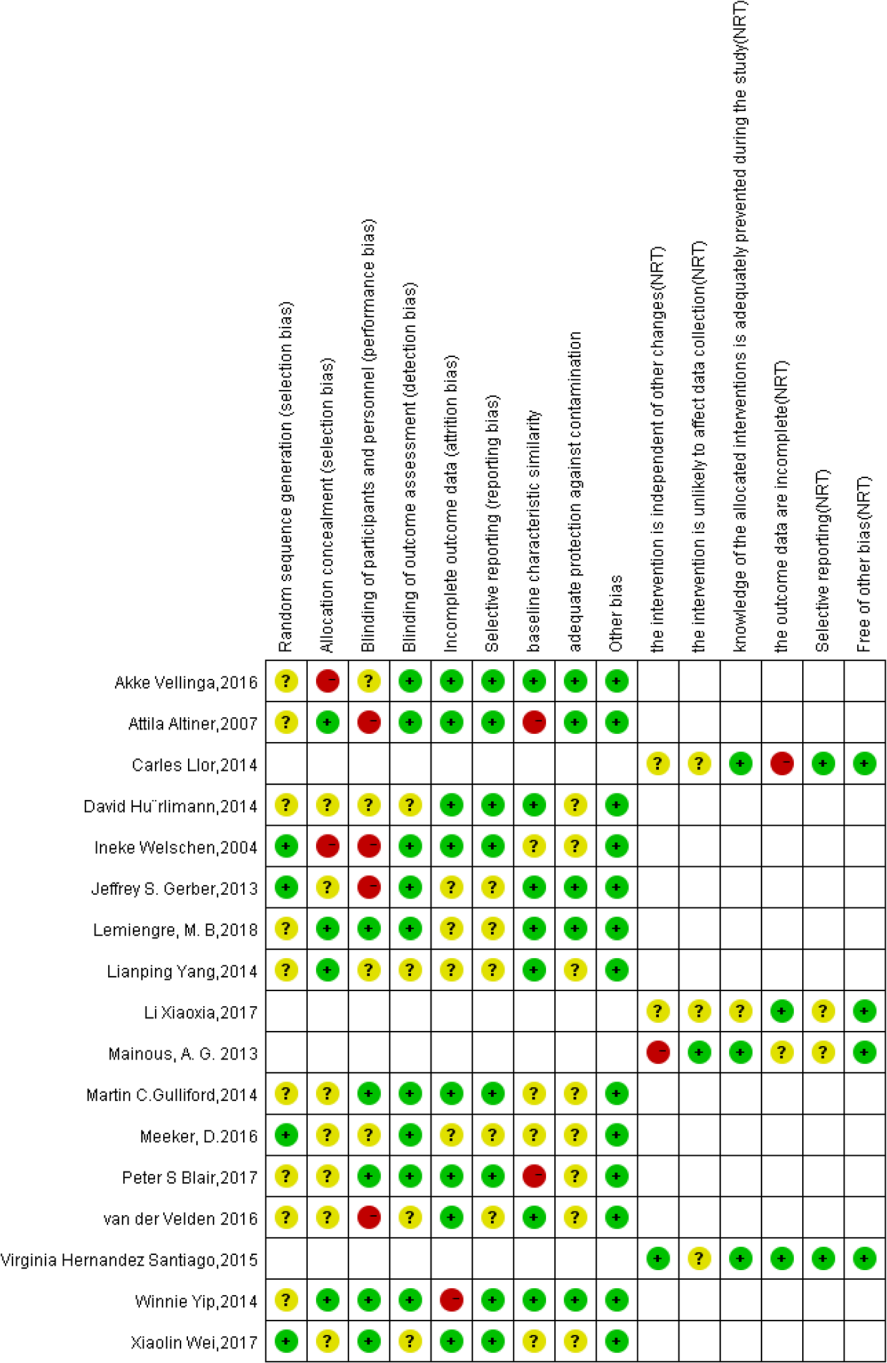
Risk of bias summary: the yellow circles mean the unclear risk of bias or the author did not mention the bias, the green circles mean the low risk of bias, the red circles mean the high risk of bias

The risk of bias was considered low if all criteria were scored as low, medium if less than three criteria were scored as medium or high, and high if more than three criteria were scored as medium or high [30]. For 13 RCTs, the risk of bias was medium for four studies [16, 18, 23, 26] and high for nine studies [14, 17, 19–22, 25, 28, 29]. The main risk of the studies was that we did not know how the random sequence generated, and the blinding of participants and personnel. For the four NRTs, the risk of the bias was medium in one study [15], high for three studies [13, 24, 27]. And the main risk was the interventions were not independent of other changes. (Figs. 2 and 3).

### Effect of interventions

Our research found that 11 of the 17 studies reported reductions of antibiotics prescribing rate between the two arms with the largest effect size reaching 29% of antibiotics prescribing. This was for upper respiratory tract infections (URTIs) in children. Three studies found improvement in in providers’ prescribing behaviours according to their guidelines. The other three studies did not find a significant difference in the prescribing rate between the intervention and the control group. Only three studies reported the sustainable effect of the interventions.

The findings of all included studies measuring the changes in antibiotics prescribing are summarized in Table 2.

**Table 2:** Antibiotic prescribing changes among these included studies

First author, year	Primary outcome(s)	Change in intervention group	Change in control group	Effect size (95% CI)	*P* value
**Educational interventions**					
Llor et al. 2014 [13]	change in the odds ratio of antibiotic prescribing (full intervention group)			0.50 (0.44 to 0.57,)	p < 0.001
	change in the odds ratio of antibiotic prescribing (partial intervention group)			0.99 (0.89 to 1.10)	NR
Wei et al. 2017 [14]	Antibiotic prescription rate	-42%	-5%	-29%	<0.001
	the multiple antibiotic prescription rate	-6%	6%	1%	0.57
	the broad-spectrum antibiotic prescription rate	-10%	-5%	-4%	0.3
	the intravenous antibiotic prescription rate	-6%	0	-8%	0.07
Hernandez Santiago et al. 2015 [15]	the rate per 1000 registered patients dispensed one or more 4C antimicrobial prescriptions after 6 months of the intervention			-33.5% (–26.1% to –40.9%)	NR
	After 12 months of the intervention			-42.2%(–34.2% to –50.2%)	NR
	After 24 months of the intervention			-55.5%(–45.9% to –65.1%)	NR
Hürlimann et al. 2014 [17]	The percentage of prescriptions of penicillins for all treated RTIs	11.8%	0.7%	11.1%	0.01
	the percentage of trimethoprim/ sulfamethoxazole prescriptions for all uncomplicated lower UTIs treated with antibiotics	13.3%	2.7%	10.6%	0.01
Lemiengre et al. 2018 [16]	Change in immediate antibiotic prescribing (intervention group of POC CRP vs. control)			1.01(0.57 to 1.79)	<0.1
	Change in immediate antibiotic prescribing (intervention group of BISNA vs. control)			2.04 (1.19 to 3.50).	<0.1
	Change in immediate antibiotic prescribing (intervention group both POC CRP and BISNA vs. control)			1.17 (0.66 to 2.09)	<0.1
**Audit and feedback interventions**					
Altiner et al. 2007 [18]	the ORs for the prescription of an antibiotic (after 6 weeks of the intervention)	0.58 (0.43 to 0.78), p<0.001	1.52(1.19 to 1.95), p=0.001		
	the ORs for the prescription of an antibiotic (after 12 months of the intervention)	0.72 (0.54 to 0.97), p=0.028	1.31(1.01 to 1.71), p=0.044		
Welschen et al. 2004 [20]	Antibiotic prescription rates for acute symptoms of the respiratory tract	-4%	8%	-12%	<0.05
Gerber et al. 2013 [19]	Rates of broad-spectrum antibiotic prescribing for bacterial ARTIs	-13%	-6%	-7%	=0.1
van der Velden et al. 2016 [21]	changes in dispensed antibiotics/1000 registered patients (first year)	-7.6%	-0.4%	-7.2%	=0.002
	changes in dispensed antibiotics/1000 registered patients (second year)	-4.3%	2%	-6.3%	=0.015
**Health policy change strategies**					
Xiaoxia 2017 [24]	changes in types of antibiotics				<0.01
	changes in drug administration of antibiotics				
	changes in combined application of antibiotic				
Yip et al. 2014 [23]	Change in antibiotic prescription rates at township health centers	:6.6%	8.4%	-15%	<0.05
	Change in antibiotic prescription rates at village posts	-6.0%	10%	-16%	<0.05
Yang 2014 [22]	Percentage of prescriptions requiring antibiotics for upper respiratory tract infections;	-3.02%;	-0.54%	-2.48%	=0.419
	Percentage of prescriptions requiring two or more antibiotics	1.93%	5.65%	-3.72%	=0.049
**Information system supported interventions**					
Gulliford et al. 2014 [25]	Proportion of consultations with antibiotics prescribed			-1.85% (0.1% to 3.59%)	=0.38
	the rate of antibiotic prescribing for respiratory tract infections			-9.69% (0.75% to 18.63%)	=0.34
Vellinga et al. 2016 [26]	proportion of antimicrobial prescribing according to guidelines for urinary tract infection (arm A vs. control)	22.8%	-1.70%	24.5%	<0.001
	proportion of antimicrobial prescribing according to guidelines for urinary tract infection (arm B vs. control)	16.7%	-1.70%	18.4%	<0.001
Blair 2017 [28]	Antibiotic prescribing rates for children’s RTIs	-12%	-21%	9%	=0.018
Mainous et al. 2013 [27]	Prescribing of broad-spectrum antibiotics rate	-16.60%	1.10%	-17.70%	<0.0001
Meeker et al. 2016 [29]	The antibiotic prescribing rate for antibiotic-inappropriate acute respiratory tract infection (intervention1 vs. control)	-16%	-11%	-5%	<0.01
	The antibiotic prescribing rate for antibiotic-inappropriate acute respiratory tract infection (intervention 2 vs. control)	-18.1%	-11%	-7.1%	<0.01
	The antibiotic prescribing rate for antibiotic-inappropriate acute respiratory tract infection (intervention3 vs. control)	-16.3%	-11%	-5.3%	<0.01

#### Educational interventions

Five studies used education interventions and out of these 5 studies? four studies reported improvements in providers’ behaviours of antimicrobial prescribing. Most educational interventions were multifaceted and included clinical guidelines, distribution of educational materials to prescribers to support clinical care, courses, workshops, conferences or other educational meetings. The greatest improvement was one ITS study reported by Virginia Hernandez who evaluated the use of educational material in British general practices regarding the of antimicrobials [15]. It was observed that after 6, 12 and 24 months, there was a highly significant and sustained decrease in 4 antimicrobials prescribing, by 33.5% (95% CI − 26.1 to − 40.9), 42.2% (95% CI − 34.2 to − 50.2) and 55.5% (95% CI − 45.9 to − 65.1) respectively (*P* value was not reported). Three RCT studies were done in China, Belgium and Switzerland. The Chinese study had an intervention effect of − 29% (95% CI − 42 to − 16; *p* = 0·0002), on antibiotics prescribing rate between the intervention group and the control group [14]. The Swiss study recommended to increase? the use of antibiotics (penicillins) for RTIs and UTIs with an effect of 11.1% (*P* = 0.01) [17].A UK study found that point-of-care C-reactive protein test without guidance is not an effective strategy to reduce antibiotics prescribing (AOR:1.01(0.57 to 1.79)*P* < 0.1) [16]. A before and after quality assurance study in Spain [13] show that the full intervention and partial intervention group both received the educational interventions according to RTI guidelines, however the full intervention group has an educational workshop on rapid tests. The study shows that the full intervention group had a lower odds ratio of antibiotics prescribing of 0.50 (95% CI: 0.44–0.57, *p* < 0.001) compared to partial intervention group 0.99 (95% CI: 0.89–1.10).

#### Audit and feedback interventions

Audit and feedback refer to a summary of health workers’ performance over specified period of time. This feedbacl is given to them in a written electronic or verbal format, including also in the form of peer review interventions. Four studies evaluated the effects of audit and feedback in primary healthcare providers. All these studies were RCTs. It was noted that all audit and feedback interventions had a positive effect in promoting rationale antibiotics prescribing.

A cluster-RCT study in Germany [18] enrolled 104 general practitioners (GPs) to receive an intervention. This intervention was visit by peers and it was focused on the communication related to antibiotics prescribing?. After the intervention, it was observed that the absolute reduction in prescribing of antibiotics was 11.7% (*P* < 0.001) and 9.8% (*P* = 0.001) after 6 weeks and 12 months of the intervention. Jeffrey S. Gerber also evaluated the effect of audit and feedback interventions among primary health care pediatricians on the use of broad-spectrum antibiotics prescribing [19]. They observed that broad-spectrum antibiotics prescribing in pediatric primary health care practices decreased from 26.8 to 14.3% among intervention group. This is when compared with the control from 28.4 to 22.6% (*P* = 0.1).

A study in Netherlands reported that the prescription rates for acute symptoms of the respiratory tract in the intervention group fell from 27 to 23%, whereas the control group rose from 29 to 37% (*P* < 0.05) [20]. Another study in Netherlands aimed to improve antibiotics prescribing quality by audit/feedback intervention. This was embedded in the primary health care practice [21]. The significant differences were observed between intervention and control practices in the changes in dispensed antibiotics/1000 registered patients (first year: 27.6% versus 20.4%, *P* = 0.002; second year: 24.3% versus + 2%, *P* = 0.015),

#### Health policy change strategies

Three policy change interventions were implemented in Chinese rural areas, two of which are matched-pair cluster-randomized trials and one was before and after study. All health policy change interventions had a positive effect in promoting antibiotics prescribing behaviors.

One policy intervention in Ningxia province changed New Cooperative Medical Scheme (NCMS) payments from fee-for-service to a capitated budget with pay-for-performance at township health centers and village posts [23]. And results suggested that capitation with pay-for-performance led to a reduction in approximately 15% in antibiotics prescriptions (*P* < 0.05).

Another matched-pair cluster-randomized trial was undertaken in Hubei province [22]. They PR (public reported) indicators about physicians’ antibiotics prescribing like percentage of prescriptions requiring antibiotics. This intervention resulted in a 9 percentage (95% CI − 17 to − 1%) reduction in the use of oral antibiotics (adjusted RR =39%, *P* = 0.027).

Another study was done in Zhejiang province, China, and it was a control before and after study [24]. They took the antibiotic prescribing as an important indicator of physicians’ professional promotion and bonus performance. They found that the outcomes of combined application of antibiotics decreased by 9.89% (*P* < 0.05) and the use of antibiotics for injection reduced to 11.42% (*P* < 0.05) at primary health care outpatient.

#### Information system supported interventions

out of 5 information supported interventions studies, in 3 studies it was observed that had a positive effect on promoting antibiotics prescribing behavior in primary health care providers.

A study including 603,409 patients [25] evaluated the effectiveness of electronically delivered decision support tools at reducing antibiotics prescribing for RTIs, and reported a reduction in antibiotics prescribing 1.85% (95% CI, 0.10–3.59%, *P* = 0.38). A quasi experimental design [27] study with nine intervention practices and 61 control practices in the Practice Partner Research Network used CDSS (clinical decision support system) intervention. A CDSS embedded in an EHR(electronic health record)resulted in a substantial decrease of 17.7% (*P* < 0.0001) on changing the overall prescribing of broad-spectrum antibiotics (e.g. macrolide antibiotics) among pediatric and adult patients. The study used suggested alternatives and accountable justification based on EHR [29], and peer comparison interventions. They reported that accountability and peer comparison as behavioral interventions resulted in reducing inappropriate antibiotics prescribing for RTIs. The antibiotics prescribing rates for antibiotics-inappropriate acute respiratory tract infection decreased 5, 7.1 and 5.3% respectively in intervention 1,2 and 3 group(*P* < 0.01) as compared to control group.

A cluster-RCT of 30 practices in Irish general practices integrated a reminder in their patient management software [26]. As a result an increase was observed in antimicrobial prescribing for urinary tract infections in the intervention arm (arm A increased 24.5%, *P* < 0.001 and arm B increased 18.4%, *P* < 0.001) relative to control arm [26]. Another cluster randomized controlled trial in England [28] used a web-based clinician-focused clinical rule to reduce antibiotics prescribing for children. The author reported that the prescribing rates among intervention arm decreased 12% as compared to control group 21% (*P* = 0.018).

## Discussion

### Main findings of this study

Most studies had a low or medium quality, indicating to have better quality design. This review found evidence that interventions of educational, audit and feedback, policy change interventions and information system reminders could promote the rational use of antibiotics in primary healthcare settings. It was observed that only three studies did not report a reduction in antibiotics prescribing rates. Educational interventions could achieve significant reductions in antibiotics prescribing by combining with other strategies including financial incentives or providing rapid C-reactive protein tests. The policy change interventions were more common in in low and middle income countries including China, and it was found that these interventions have a good impact on decreasing the antibiotics prescribing rate. The information system supported intervention could have different outcome in different settings. However, we cannot make general recommendations to guide the selection of different interventions due to limitations in heterogeneity of the interventions.

### Findings in relation to other research

In a systematic review conducted at England of antimicrobial Stewardship in Outpatient Settings, it was observed that antimicrobial stewardship programs in outpatient settings improve antimicrobial prescribing without adversely effecting patient outcomes and [31]. These results were in line with our study indicating that primary healthcare providers’ prescription behavior interventions are associated with a reductions in antibiotics prescribing and in promoting the rational use of antibiotics. They evaluated the effectiveness of physician-targeted interventions to improve antibiotics use for respiratory tract infections, and reported a reduction of 11.6% of antibiotics prescription [32]. Another review shows that just developing guidelines is not enough to restrict antibiotics prescribing and there is a need have educational material supplemented to with another intervention? [24]. This review found that multiple interventions aiming to improve educational material for the physician’ were most often effective [33]. For the audit and feedback interventions, our study finds this strategy is effective in promoting the antibiotics prescribing. This is also consistent with the study done by Davey P [5].

A previous systematic review has shown that computer interventions, educational sessions, collaboratively developed guidelines and training videos were effective in changing practice of pediatricians. It was also observed that multifaceted and computer interventions work best [34]. The interventions in primary health care were different from the interventions? conducted in the hospital setting. Interventions in hospitals were more systematic such as the introduction of new diagnostic tests to guide antibiotic treatment and expert audit of prescriptions and either feedback provided to prescribers on their prescribing. Primary health care providers’ interventions in outpatient usually aim to change individual prescriber’s behavior. This behavior is influenced by social norms, attitudes and beliefs [35].

### Recommendations for future research

Future research should focus on the design and methodology of high-quality RCTs. We found that there were few studies reporting the sustainable effects of the interventions [36]. In our opinion the studies should aim for longer periods of follow-up. Future studies assessing the quality of intervention and implementation are needed. Also the interventions should pay more attention towards the providers’ behaviors [37].

### Strengths and limitations

This review is vital as we evaluated the effect of primary health care providers targeted interventions and provided an evidence-base. There are many reviews on the effectiveness of antibiotics stewardship of inpatients, however, few focus on outpatients. A key strength of our review is that only studies with a control group, ITS or control before and after studies were included and therefore are more likely to represent the change.

However, there are several limitations in our review. First, we only identified studies that were published, so the results may be affected by publication bias although not all interventions were statistically significant. The effect sizes from the included studies in this review may be misleading because published trials are more likely to demonstrate positive and large intervention effects. Second, most studies identified were from the US, Europe and China which may be suggestive of the bias, however this is what is available in the literature. Third, study designs of included studies were complex and heterogeneous, making it challenging to judge the quality of these studies.

## Conclusions

Our review demonstrated that there were few studies describing antibiotics improving interventions targeted towards primary healthcare providers in LIMCs. It was challenging to compare these studies because the included studies had heterogeneous study designs and were conducted in different settings. There moderate-strength evidence shows that provider-targeted interventions can decrease the antibiotics prescribing and can promote the rational use of antibiotics. Most of the interventions had a moderate or strong effect of antibiotics prescribing reduction or promotion of antibiotics rational use.

## Supplementary information

**Additional file 1.**

## Data Availability

Lu Yao and Qiang Sun had full access to all the data in the study and take responsibility for the integrity of the data and the accuracy of the data analysis. Data will be provided under request to the first authors.

## Abbreviations

LMICs: Low-income and-Middle-Income Countries
ABR: Antibiotics Resistance
CNKI: China National Knowledge Infrastructure
PICOS: Population, Intervention, Comparison, Outcome and Study design
WHO: World Health Organization
EPOC: Effective Practice and Organisation of Care
RCTs: Randomized Controlled Trials
ITS: Interrupted Times Series
NRT: Non-Randomized Trial
RTIs: Respiratory Tract Infections
URTIs: Upper Respiratory Tract Infections
GPs: General Practitioners
NCMS: New Cooperative Medical Scheme
PR: Public Reported
CDSS: Clinical Decision Support System
EHR: Electronic Health Record
