# Supplementary material for: The effects of the primary health care providers’ prescription behavior interventions to improve the rational use of antibiotics: a systematic review
Source: Glob Health Res Policy. 2020 Oct 17;5:45. doi: 10.1186/s41256-020-00171-2 (PMC7568391; doi:10.1186/s41256-020-00171-2)
Supplement: Supplementary file 1 — Additional file 1. [file 41256_2020_171_MOESM1_ESM.docx]

**Search strategy**

**Ovid Medline (January 1st, 1998 to December 1st, 2018)**

**Last searched 1^th^ July 2019**

**Limits: Humans and English language**

1. communit$.ab,ti. (727595)
2. primary health.ab,ti. (28433)
3. (primary care or ambulatory care or office visits or general practice or family medicine or family practitioner).ab,ti. (159182)
4. (outpatient or outpatients).ab,ti. (228766)
5. 1 or 2 or 3 or 4 (1088228)
6. antibiot$.ab,ti. (404048)
7. antimicrob$.ab,ti. (194034)
8. antibacteria$.ab,ti. (81709)
9. 6 or 7 or 8 (596333)
10. steward$.ab,ti. (10233)
11. educat$.ab,ti. (812726)
12. (audit and feedback).ab,ti. (3310)
13. health policy change strategies.ab,ti. (0)
14. information system.ab,ti. (24697)
15. (intervention or interventions or interventional).ab,ti. (1341386)
16. 10 or 11 or 12 or 13 or 14 or 15 (2058993)
17. 5 and 9 and 16 (4720)
18. limit 17 to (English language and humans) (2326)

**Web of science (January 1st, 1998 to December 1st, 2018)**

**Last searched 1^th^ July 2019**

**Limits: English language**

#1 TS=(community OR (primary health) OR outpatient ), (2,443,381)

#2 TS=(primary care or ambulatory care or office visits or general practice or family medicine or family practitioner).(355147)

#3 TI=(antibiotic OR antimicrobial OR antibacterial), (301,472)

#4 TS=(stewardship OR education OR (audit and feedback) OR (health policy) OR (information system) OR intervention ), (3,458,794)

#5 (1 AND #2 AND #3 AND#4) AND LANGUAGE: (English) (2,252)

**Cochrane Library (January 1st, 1998 to December 1st, 2018)**

**Last searched 1^th^ July 2019**

**Limits: None**

#1 community. ti,ab,kw (40754)

**#2** primary health ti,ab,kw (69240)

**#3**(primary care or ambulatory care or office visits or general practice or family medicine or family practitioner). ti,ab,kw (88794)

**#4** #1 or #2 or #3 (104010)

**#5** (antibiotic or antimicrobial or antibacterial). ti,ab,kw (38585)

**#6 stewardship** ti,ab,kw (316)

**#7 (education or educational)** ti,ab,kw(72255)

**#8** (audit and feedback) ti,ab,kw (768)

**#9 (**health policy change strategies**)** ti,ab,kw **(343)**

**#10 (**information system**)** ti,ab,kw (27543)

**#11** (intervention or interventions or interventional) ti,ab,kw (351652)

**#12** #5 or #6 #7 or #8 or #9 or #10 or #11 (485234)

**#13** #4 and #10 (532)

CNKI **(January 1st, 1998 to December 1st, 2018)**

**Last searched 1^th^ July 2019**

**Limits: None**

1. ‘prescription’ ti.(118153)
2. ‘community’ or ‘primary health’ or ‘outpatient’ or ‘rural doctors’ or ‘village doctors’ ti. (569118)
3. ‘intervention’ ti.(364743)
4. ‘antimicrobial’ or ‘antibacterial’ or ‘antibiotic’ ti. (139032)
5. 1 and 2 and 3 (587)
